# Comprehensive Evaluation of Long-Term Dentin Bond Strength, Water Sorption, Solubility, and Degree of Conversion of Self-Adhesive Resin Composites

**DOI:** 10.3290/j.jad.b5749506

**Published:** 2024-09-17

**Authors:** Ye Yao, Di Wu, Carolina Cecilia Cifuentes-Jimenez, Hidehiko Sano, Pedro Alvarez-Lloret, Monica Yamauti, Atsushi Tomokiyo

**Affiliations:** a PhD Student, Department of Restorative Dentistry, Graduate School of Dental Medicine, Hokkaido University, Kita 13 Nishi 7, 060-8586, Sapporo, Japan. Conceptualization, methodology, formal analysis, investigation, data curation, writing (original draft).; b PhD Student, Department of Restorative Dentistry, Graduate School of Dental Medicine, Hokkaido University, Kita 13 Nishi 7, 060-8586, Sapporo, Japan. Investigation, data curation, formal analysis, review, and editing.; c Post-doctoral Fellow, Department of Stomatology, Faculty of Dentistry, University of Granada, Campus de Cartuja, s/n, 18011, Granada, Spain. Investigation, methodology, data curation, formal analysis, review, and editing.; d Emeritus Professor, Department of Restorative Dentistry, Graduate School of Dental Medicine, Hokkaido University, Kita 13 Nishi 7, 060-8586, Sapporo, Japan. Resources, funding acquisition, project administration, writing (review and editing).; e Associate Professor, Department of Geology, Faculty of Geology, University of Oviedo, Campus de Llamaquique, s/n, 33005, Oviedo, Spain. Investigation, methodology, data curation, formal analysis, resources, funding acquisition, writing (review and editing).; f Associate Professor, Department of Restorative Dentistry, Graduate School of Dental Medicine, Hokkaido University, Kita 13 Nishi 7, 060-8586, Sapporo, Japan. conceptualization, resources, data curation, formal analysis, supervision, validation, visualization, methodology, writing (original draft, review, and editing).; Clinical Associate Professor, Department of Biomedical and Applied Sciences, Indiana University School of Dentistry, 46202 , Indianapolis, IN, USA; g Professor, Department of Restorative Dentistry, Graduate School of Dental Medicine, Hokkaido University, Kita 13 Nishi 7, 060-8586, Sapporo, Japan. Resources, writing (review and editing).

**Keywords:** self-adhesive resin composite, adhesion to dentin, physical properties, bond strength

## Abstract

**Purpose:**

To evaluate the long-term microtensile bond strength (µTBS) to dentin, water sorption (WSP) and solubility (WSL), and degree of conversion (DC) of self-adhesive resin composites (SACs).

**Materials and Methods:**

The mid-coronal dentin of human molars was exposed, and teeth were randomly assigned to five groups according to the SACs (n = 10): 1. FIT SA F03 (FIT); 2. Experimental (EXP); 3. Fusio Liquid Dentin (FLD); 4. Vertise Flow (VER); 5. Constic (CON). The µTBS was evaluated after 24 hours (24 h) and 6 months (6 m) storage. A scanning electron microscope examined failure modes and resin–dentin interfaces. The WSP and WSL (n = 5) were evaluated following ISO 4049:2019 specifications, and DC (n = 3) was measured using Raman spectroscopy. The statistical analyses were performed accepting a significance level of p = 0.05.

**Results:**

FIT, EXP, and FLD produced significantly higher µTBS median values than VER and CON after 24 h and 6 m (p < 0.05). After 6m, the µTBS median of FIT and EXP significantly decreased (p < 0.05), while FLD, VER, and CON showed no significant difference (p > 0.05). FLD and CON exhibited lower WSP than FIT, EXP, and VER (p < 0.05). FLD presented the lowest (p < 0.05), and VER revealed the highest WSL (p < 0.05). FIT and EXP showed the highest (p < 0.05), and VER demonstrated the lowest DC (p < 0.05).

**Conclusions:**

Following the present study’s design, SACs’ bonding performance and physical properties remained restricted. Therefore, the application should be considered cautiously, and further clinical trials are necessary to evaluate their long-term performance.

Direct resin composite restorations bonded to tooth structures with adhesive systems have been the preferable treatment for restoring dental caries and other hard tissue diseases during the past two decades, allowing minimally invasive dentistry management of those diseases.^21,38^ Still, resin composite restorations fail mainly due to caries around the restorations, fractures, and esthetic reasons.^16,34^ The advancements in adhesive dentistry, such as the development of universal adhesives, self-adhesive resin cements, and self-adhesive restorative resin composites, have provided simplified, faster, and less technique-sensitive restorative procedures.

The first self-adhesive material, resin cement,^19^ was introduced in the early 2000s. Self-adhesive resin cements are luting agents based on filled polymers that bond to tooth structure without utilizing a separate adhesive or etchant, combining the advantages of adhesives and conventional luting agents.^19^ A similar rationale has been used to develop restorative resin composites. The first self-adhesive flowable resin composite was also produced in the 2000s. Self-adhesive flowable resin composites (SACs) combine the components of the self-etching adhesive system and flowable resin composite in a single formulation, eliminating the need for an additional adhesive application step. Applying SACs would simplify the restorative procedure, decrease the dentist’s technical sensitivity, and save chairside time.^47^ Although the manufacturers claim simplified handling, the bond durability and stability should be addressed before endorsing this clinical application.^25^

SACs mainly contain acidic functional monomers (eg, 10-methacryloyloxydecyl dihydrogen phosphate, glycerol phosphate dimethacrylate and 4-methacryloxyethyl trimellitic acid) for etching dental surfaces and 2-hydroxyethyl methacrylate (HEMA) to enhance dentin wettability and resin permeability. The bonding of SACs is achieved through the chemical bonding of the acidic functional monomer to the calcium ions present in the hydroxyapatite structure and the micromechanical locking of polymer monomers to demineralized hard tissues.^45^ However, if SACs are not adequately polymerized, they may release unreacted or partially reacted monomers, filler particles, degradation products, and other additives in the oral environment.^45^ Consequently, the eluted monomers could also affect the structural stability of the material and longevity in the oral cavity.^2^

Although dental manufacturers have offered several SACs for clinical use, more research needs to be done on their bonding performance and other physical properties affecting their application. Previous studies have shown that SACs had lower short-term bond strengths than conventional resin composites utilizing etch-rinse or self-etch adhesives.^11,35^ However, the long-term bond strength data^7,8,25,40^ and clinical trials using those materials for restorations^15,28,39^ are scarce. Although the application of SACs is relatively simple, previous studies of first-generation SACs have shown that their adhesive and physical properties were inferior to those using adhesives.^1,17,36^ In addition, some clinical studies demonstrated that SACs were acceptable as Class I restorations and pit and fissure sealants,^14,39^ and one SAC was found unacceptable.^9^ Consequently, physicians have remained resistant to eliminating the adhesive step from the restorative process.^11^

Therefore, this study aimed to comparatively evaluate four commercial and one experimental SACs according to (I) bond strength after 24 h and 6 m of storage in distilled water, (II) water sorption and solubility, and (III) degree of conversion. The null hypotheses were that (i) storage time would not affect SACs’ bond strength to dentin; (ii) different SACs would not affect bond strength to dentin; (iii) different SACs would not interfere in their water sorption and solubility; and (iv) degree of conversion would not be affected by different materials.

## Material and Methods

### Study Design

This was a quantitative, qualitative, and prospective laboratory study. The independent variables were self-adhesive resin composite materials (5 levels) and the dependent variables were microtensile bond strength (µTBS) to dentin, failure mode, water sorption (WSP) and solubility (WSL), and degree of conversion (DC). The variable “storage time” (two levels) was tested for the µTBS outcome.

### Tooth Preparation for µTBS Test

One hundred and thirty sound human third molars were collected with the patient’s informed consent and approved by the local Ethics Committee (protocol #2018–9). All extracted teeth were cleaned and stored in a 0.5 wt% chloramine-T solution at 4°C and used within six months after extraction. The teeth and the SACs used in the study were removed from the refrigerator and kept at room temperature for at least 30 min before testing. All five SACs were tested in the shade A3 and the details of the SACs and their application modes are shown in Table 1.

**Table 1:** Resin composite materials used in this study

Resin composite	Abbreviation	Manufacturer	Lot no.	Composition	Filler load (wt%)	Manufacturer’s instructions for use
FIT SA™ F03 (Shade A3)	FIT	Shofu, Kyoto, Japan	052004	UDMA, HEMA, phosphonic acid monomer, S-PRG filler based on fluoroboroaluminosilicate glass, zirconium silicate filler	68.2	Dry the tooth surface with air to remove excess moisture. Do not desiccate the cavity surface. Dispense the material into the prepared cavity and spread uniformly onto the cavity walls and floor (film thickness <0.5 mm) with the needle tip, hand instrument, or gently air blow as desired. Leave it undisturbed for 20 s and remove the excess of material. Light cure each layer for 5 s with an LED light-curing unit (wavelength: 440–490 nm; irradiance: ≥ 1000 mW/cm^2^). Subsequently, apply the material in increments of 2 mm thickness or less and cure each layer for 10 s.
Experimental (Shade A3)	EXP	Shofu, Kyoto, Japan	HFS F03	UDMA, HEMA, phosphonic acid monomer, silica filler, zirconium silicate filler	67.9	Dry the tooth surface with air to remove excess moisture. Do not desiccate the cavity surface. Dispense the material into the prepared cavity and spread uniformly onto the cavity walls and floor (film thickness <0.5 mm) with the needle tip, hand instrument, or gently air blow as desired. Leave it undisturbed for 20 s and remove the excess of material. Light cure each layer for 5 s with an LED light-curing unit (wavelength: 440–490 nm; irradiance: ≥ 1000 mW/cm^2^). Subsequently, apply the material in increments of 2 mm thickness or less and cure each layer for 10 s.
Fusio™ Liquid Dentin (Shade A3)	FLD	Pentron Clinical, Orange, CA, USA	8184167	4-MET, UDMA, TEGDMA, HEMA, amorphous silicon nanosized, silanized Ba-glass	65	Apply the material in 1 mm increments. Agitate for 20 s with the needle tip or a brush. Light cure for 10 s. Apply additional 2 mm increments. Light cure each increment 10 s. Light cure the final increment and additional 10 s.
Vertise™ Flow (Shade A3)	VER	Kerr, Brea, CA, USA	8315394	GPDM, HEMA, Bis-GMA, catalysts, prepolymerized filler, barium glass filler, colloidal silica, ytterbium fluoride	70	Wash the tooth surface thoroughly with water spray and air dry at maximum air pressure for 5 s. Dispense the material onto cavity with provided dispensing tip. Use provided brush to apply the material to the entire cavity wall with moderate pressure for 15–20 s to obtain a thin layer (<0.5 mm). Note: Replace syringe cap after each use to prevent the resin from hardening in the syringe. Light cure for 20 s. Layer the material with increments of 2 mm or less. Light cure each increment for 20 s.
Constic (Shade A3)	CON	DMG Chemisch-Pharmazeutische Fabrik, Hamburg, Germany	243895	10-MDP, Bis-GMA, EBADMA, UDMA, HEMA, TEGDMA, HDMA, Ba-glass	65	Dry the tooth using water- and oil-free air in order to avoid overdrying of the dentin. A moist layer must remain on the surface of the tooth. Apply the material onto the cavity surface with the aid of the Luer-Lock-Tip by pressing the syringe and massage a thin layer (≈ 0.5 mm) into the entire surface of the cavity wall for 20 s using the brush. Light cure for 20 s. Layer the material with a maximum 2 mm layer thickness and cure each layer for 20 s. Note: It is not necessary to repeat massaging of the individual layers.

UDMA, urethane dimethacrylate; HEMA, 2-hydroxyethyl methacrylate; TEGDMA, triethyleneglycol dimethacrylate; 4-MET, 4-methacryloxyethyl trimellitic acid; GPDM, glycerol phosphate dimethacrylate; Bis-GMA, bisphenol A diglycidyl ether dimethacrylate; EBADMA, ethoxylated bisphenol A dimethacrylate; HDMA, 1,6-hexanediol dimethacrylate; 10-MDP, methacryloyloxydecyl dihydrogen phosphate

The occlusal enamel was removed to expose the flat mid-coronal dentin surface using a model trimmer (Model Trimmer MT 10, J. Morita MFG, Tokyo, Japan) and standardized smear layers were produced with 600-grit silicon carbide paper under running water.^4^ The teeth were randomly divided into five groups according to the SACs, which were applied on the dentin surfaces following the manufacturer’s instructions (Table 1). The first increment had an approximate thickness of 0.5 mm, and the following increments had a maximum of 2.0 mm, as recommended by the manufacturers (Table 1). Each increment of each resin composite was light-cured using a blue LED light-curing unit (Pen Cure 2000 VL-10, J. Morita MFG, Tokyo, Japan, light irradiance = 2000 mW/cm^2^) according to the manufacturers’ recommendations to form the resin blocks (4 mm height). The prepared teeth were kept in distilled water at 37°C for storage. Each group was randomly assigned to short-term [24 hours (24 h), n = 10] and long-term [6 months (6 m), n = 10] storage for the subsequent µTBS test. During the 6-m storage period, the medium was renewed weekly with distilled water.

### µTBS Test and Failure Mode Analysis

After storage, resin–dentin bonded beams (cross-sectional area: 1 mm^2^) were obtained by sectioning the restored teeth longitudinally in both directions (“x” and “y”) using a low-speed diamond saw (Isomet 1000, Buehler, Lake Bluff, USA) according to the non-trimming technique.^4^ Subsequently, each bonded beam was affixed to Ciucchi’s jig with a cyanoacrylate adhesive (Model Repair II Blue, Dentsply-Sankin, Tokyo, Japan) and placed in an EZ-S test device (Shimadzu, Tokyo, Japan). Bonded beams were subjected to tensile force employing a 500-N load cell at a 1 mm/min crosshead speed in a desktop testing apparatus (EZ-S, Shimadzu, Kyoto, Japan) until fracture occurred. Each beam was tested within 5 min after removal from water storage and protected from drying until testing. Each beam’s tensile load causing fracture was recorded and divided with the cross-sectional area to obtain the µTBS in megapascals (MPa). The bond strength values of all beams from the same tooth were averaged and the tooth served as the statistical unit.^4^ Following the µTBS test, the failure modes were observed with a stereomicroscope (SMZ-171-TLED, Shimadzu, Kyoto, Japan) at 50× magnification and were classified into interfacial failure between dentin/resin composite (adhesive failure), cohesive failure exclusively in dentin (dentin failure) or resin composite (composite failure), and mixed adhesive-cohesive failure (mixed failure). In addition, fractured ends of resin composite and dentin were inspected using a scanning electron microscope (SEM; S-4800, Hitachi, Tokyo, Japan). The fracture ends were attached to an aluminum stage and coated with platinum-palladium for 120 seconds using an ion sputter (E-1030, Hitachi, Tokyo, Japan). Observations of failure modes were performed at the voltage of 10 kV.

### Morphology of Resin–Dentin Interfaces

Additional teeth were prepared and stored, as previously described, for resin–dentin interface observations (n = 3).^22^ After 24 h and 6 m, they were sectioned longitudinally to the long axis to obtain resin–dentin slices (2 mm thick) using a low-speed diamond saw (Isomet 1000, Buehler, Lake Bluff, USA). The slices were sequentially polished under copious water with #600, #800, and #1000 grit silicon carbide paper and 6, 3, 1, and 0.25 µm diamond pastes (DP-Paste P, Struers, Denmark) for 60 s each paper or paste preparation. Ultrasonic cleaning was done after each polishing step for 3 min. The polished slices were treated with 1 M hydrochloric acid solution for 10 s and then into 5% sodium hypochlorite solution for 5 min. Abundant water irrigation was done after both treatments. The slices were removed from the solution and dried overnight under ambient conditions, ion-sputtered coated with Pt-Pd for 120 s, and the morphology of resin–dentin interfaces was observed with SEM at 10 kV.

### Measurement of WSP and WSL

Disc-shaped samples of each SAC were prepared following the ISO 4049:2019 specification to evaluate WSP and WSL (n = 5), using a split-metal mold of internal dimensions 15.0 ± 0.1 mm in diameter and 1.0 ± 0.1 mm thick. A polyester film was placed on the bottom of the metal mold, the SACs were inserted, and another layer of polyester film was placed on top of SACs. The whole set was covered with a glass slide to remove the excess material. The samples were cured with an 8 mm tip LED light-curing unit (Pen Cure 2000 VL-10, J. Morita MFG, Tokyo, Japan, light irradiance = 2000 mW/cm^2^) for 40 s on each surface (top and bottom). Each surface was irradiated four times for 10 s/irradiation with overlapping curing areas. The total irradiation time was 80 s for each sample. Then, the mold was transferred to the incubator, maintained at 37 ± 2°C for 15 min. The irregular regions on the surface of the samples were polished with #1000 grit silicon carbon paper. The diameter of the completed samples was not less than 14.8 mm.

All samples were dried in a desiccator with silica gel for 22 h at 37 ± 2°C, then for an extra 2 h in another desiccator at 23 ± 2°C. Each sample was weighed iteratively on an analytical scale daily before achieving a constant (μg, variation less than 100 μg for 3 consecutive days) mass (m^1^). Following the attainment of a continuous mass, a digital caliper was used to measure the diameter and thickness of each sample two times in mutually perpendicular positions, and the volume (V, mm^3^, V = πr^2^ h) of each sample was calculated with the mean radius (r, mm) and mean thickness (h, mm). The samples were stored in glass vials containing 20 ml of deionized water at 37°C. After 7 days, the samples were removed from the tubes, gently dried with absorbent paper, and weighed again for mass (m^2^). The samples were returned to the desiccator and weighed daily until obtaining a constant mass (m^3^, μg, variation less than 100 μg for 3 consecutive days). WSP and WSL were calculated by following the equations:

$$\frac{W_{SP}=(m^{2}-m^{3})}{V}\;\frac{W_{SL}=(m^{1}-m^{3})}{V}.$$

The ISO 4049:2019 standard was considered compliant when *W_SP_ ≤ 40 μg / mm^3^* and *W_SL_ ≤ 7.5 μg / mm^3^*.

### Degree of Conversion Analysis

A modular confocal Raman spectroscope was used to investigate the resin composites’ DC under cured and uncured conditions. Spectra were obtained using a JASCO NRS-5100 spectrometer (Jasco Inc, Easton, MD, USA) with a charge-coupled device detector (1024 × 256 pixels) cooled by a Peltier-effect module. The SAC were injected in a proper circular Teflon sample holder (10.0 mm diameter × 4.0 mm depth), which was placed under the microscope on a computer-controlled XYZ stage, focusing the laser beam with a 20× lens (Olympus optical microscope). A near-infrared diode laser (785 nm) kept at 500 mW was employed to induce the Raman scattering. Spectra were acquired between 1000 and 1800 cm^-1^ using an exposure time of 5 s and 10 accumulations with an average spectral resolution of 1.6 cm^-1^. Subsequently, to the uncured measurements, the samples were cured with Pen Cure 2000 VL-10 LED light-curing unit following the manufacturer’s recommended curing time, and the cured measurements were taken. Instrument calibration was determined before data acquisition by comparison with the spectrum of silicon standard to set the reference position at 520 cm^-1^.

Three samples were employed for spectral analyses for each material. The DC values were calculated by determining the polymerized samples in terms of the changing peak amplitude ratio of the absorbance aliphatic C=C at 1638 cm^-1^ and the internal reference peak of aromatic C=C at 1608 cm^-1^.^37,43^ The intensity/amplitude of the reference peaks were obtained to determine possible differences due to scattering in the Raman spectrum.^6^ A region of the spectra between 1590 and 1660 cm^-1^ was selected and baseline corrected; after spectrometric analyses, the DC was calculated as follows:

$$\text{DC }{\left(\%\right)}_{amplitude/intensity}=[1-\frac{Cured\;C=C\;1638\;cm^{-1}/Cured\;C=C\;1608\;cm^{-1}}{Uncured\;C=C\;1638\;cm^{-1}/Uncured\;C=C\;1608\;cm^{-1}}]\times 100.$$

Amplitude/intensity values were resolved using curve-fitting software Peakfit v4.12 (Systat Software, Chicago, IL, USA). The second derivative method was used for peak measurements within the spectral region. The degree of smoothing was set at 20% (Savitzky–Golay algorithm) and a mixed Gaussian-Lorentzian function was employed to fit the peak profiles (ie, curve shape and width). Curve fitting was accepted when r^2^ reached values up to 0.995.

### Statistical Analysis

Data were analyzed with the software SPSS version 26 (Statistical Package for the Social Sciences; Chicago, IL, USA). For the µTBS test, the statistical unit was “tooth” and the pre-testing failure value was recorded as 0 MPa. µTBS data did not follow normal distribution and variance homogeneity and were analyzed using the Kruskal–Wallis and Bonferroni correction for multiple tests. WSP data followed a normal distribution, but the variance was not homogeneous; thus, it was analyzed using Welch’s ANOVA and Tamhane test. WSL and DC data followed normal distribution and variance homogeneity. WSL data were analyzed using One-way ANOVA and Bonferroni test. The DC data was analyzed using One-way ANOVA and the Holm-Sidak test. The significance level for all statistical analyses was set at α < 0.05.

## Results

### µTBS and Failure Mode

**Fig 1:**
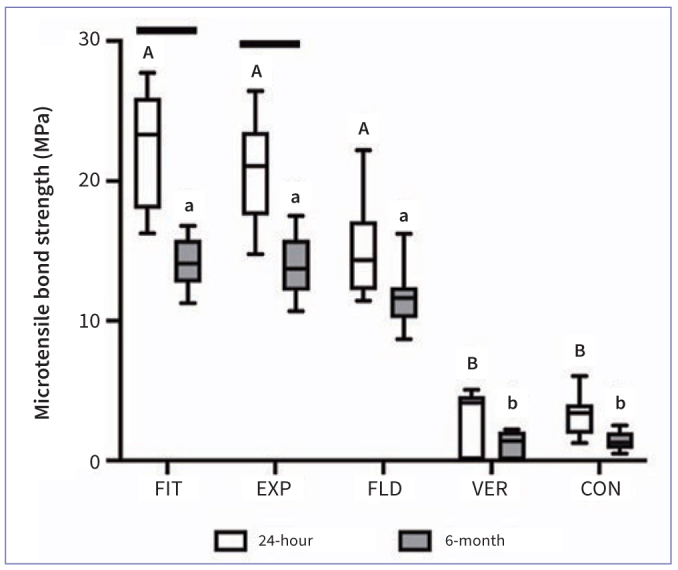
Results of microtensile bond strength (µTBS) of dentin–resin composite samples after 24 h and 6 m of water storage. Different capital letters represent statistically significant differences in the bond strength between the self-adhesive resin composites at 24 h (p < 0.05). Different lowercase letters indicate statistically significant differences in the bond strength between the self-adhesive resin composites at 6 m (p < 0.05). Bars connecting 24 h and 6 m results depict statistically significant differences (p < 0.05).

**Fig 2:**
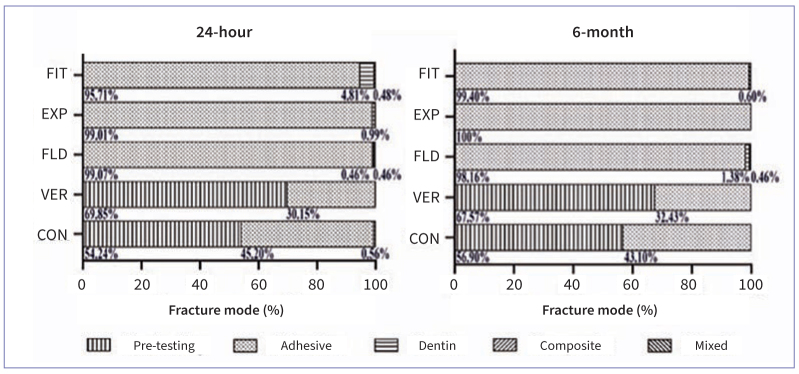
Distribution of failure modes of dentin–resin composite specimens after 24 h and 6 m of storage, including the pre-testing failures. The numbers below each bar segment indicate the percentage value of the corresponding type of failure.

**Fig 3:**
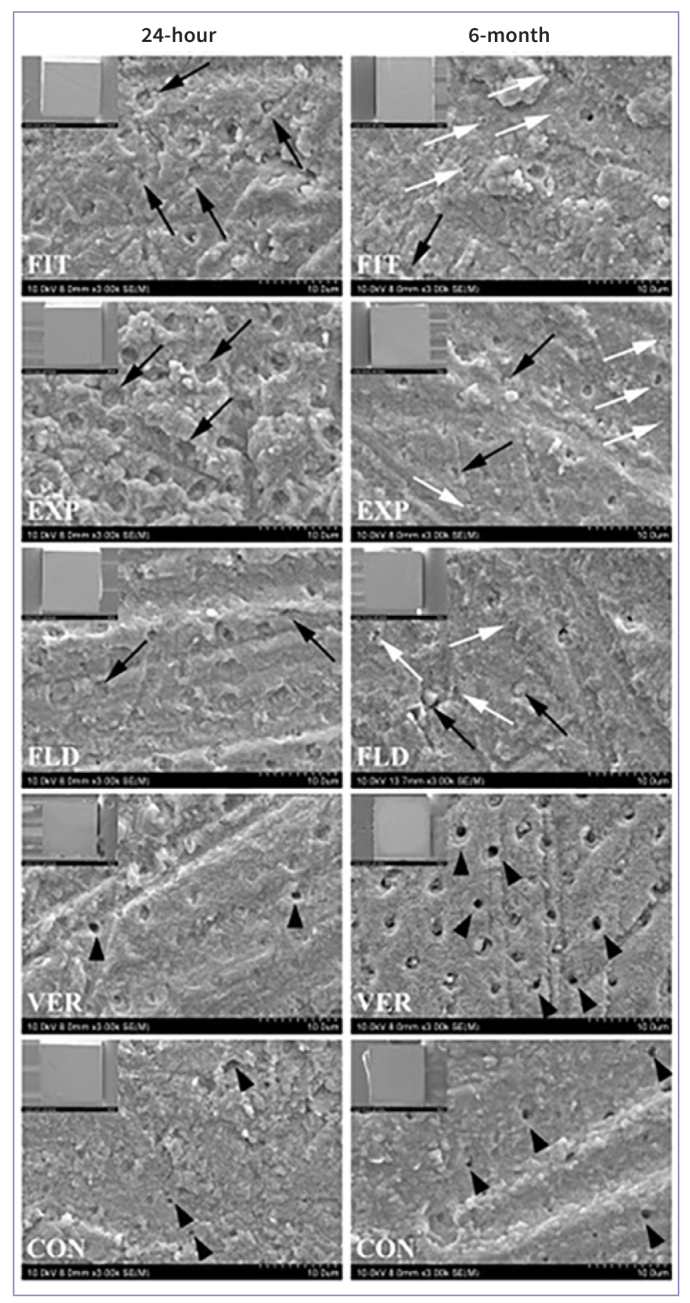
Representative SEM images of each group’s dentin side of adhesive failure after 24 h and 6 m (magnifications: 80× [small images on the top left] and 3000× [large images]). After both storage periods, resin obliterating dentin tubules (black arrows) could be observed in FIT, EXP, and FLD, indicating residues of self-adhesive resin composites on the dentin surface. Some small round voids (white arrows) could indicate air or water entrapped in FIT, EXP and FLD after 6 m storage. Also, open dentin tubules were mainly detected in VER and CON (black arrowheads), which could indicate the debond of self-adhesive resin composites from the dentin surface.

The results of microtensile bond strength are expressed as a median in Figure 1 and Table 2, which depicts that distributions of µTBS were not similar for all groups. Pre-testing failure was commonly observed in VER and CON and a “zero” value was attributed to each pre-testing failed resin–dentin beam. The µTBS were statistically significantly different between groups, χ^2^(9) = 80.800, p < 0.001. Pairwise comparisons using Bonferroni correction for multiple tests showed that, after 24 h storage, FIT, EXP, and FLD showed significantly higher µTBS than VER and CON (p = 0.000). After 6 m, the µTBS of FIT and EXP significantly decreased (p = 0.016), and there was not a significant difference in µTBS of FLD (p = 0.328), CON (p = 1.000), and VER (p = 1.000). Also, FIT, EXP, and FLD exhibited significantly higher µTBS than VER and CON (p = 0.000) after 6 m storage. The distribution of the failure mode is shown in Figure 2. The 24 h and 6 m µTBS failure mode in FIT, EXP, and FLD was predominantly adhesive (>95%). VER and CON had many pre-testing failures at 24 h and 6 m (>45%). Figure 3 shows representative SEM images of fractured dentin surfaces of adhesive failure at 80× and 3000× magnifications. After 24 h in FIT, EXP, and FLD, residues of SACs could be observed obliterating the dentin tubules, while in the VER, some dentin tubules were open (black arrowheads). After 6 m, some dentin tubules were clogged by resin (black arrows) in FIT, EXP, and FLD and also some voids (white arrows) were noted, which could be related to the decrease in bond strength. Open dentin tubules were observed at 6 m storage in VER and CON. According to the SEM images, there seems to be a material loss from 24 h to 6 m.

**Table 2:** Summary table of microtensile bond strength statistics output (n = 10). The values of microtensile bond strength were expressed in MPa

Resin composite	Storage time	Median	Minimum	Maximum	Percentiles distributions (%)			Interquartile range
					25	50	75	
FIT	24 h	23.31	16.25	27.73	18.01	23.31	25.95	7.94
	6 m	14.09	11.26	16.78	12.73	14.09	15.78	3.05
EXP	24 h	21.08	14.75	26.43	17.53	21.08	23.51	5.98
	6 m	13.72	10.67	17.49	12.16	13.72	15.78	3.62
FLD	24 h	14.34	11.42	22.19	12.20	14.34	17.11	4.91
	6 m	11.62	8.68	16.22	10.18	11.61	12.41	2.23
VER	24 h	4.12	0.00	5.07	0.00	4.12	4.60	4.6
	6 m	1.39	0.00	2.22	0.00	1.39	2.07	2.07
CON	24 h	3.38	1.25	6.06	1.91	3.38	4.00	2.09
	6 m	1.29	0.49	2.51	0.84	1.29	2.01	1.17

### Morphology of Resin–Dentin Interfaces

**Fig 4:**
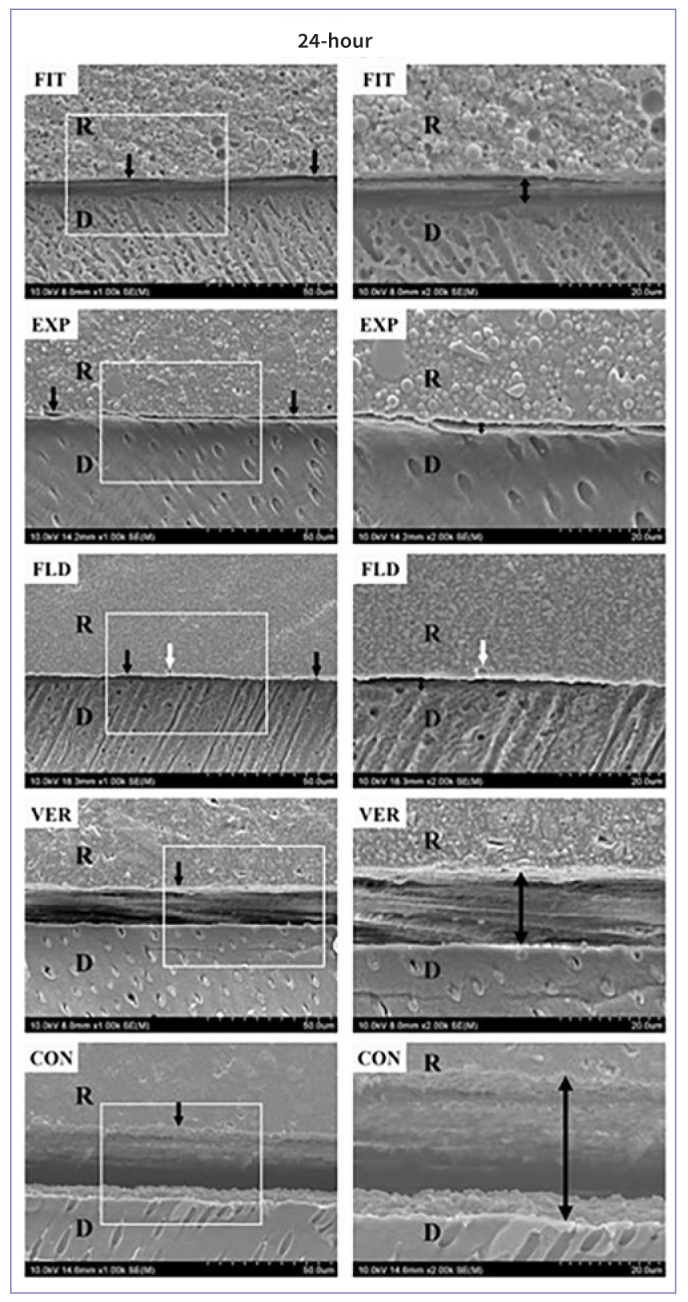
SEM observations (magnifications: 1000× [left images] and 2000× [right images]) of the dentin–resin interface of each group after 24 h storage. FIT, EXP, and FLD showed small gaps (black arrows/double-headed arrows) at the resin–dentin interface, while VER and CON showed more pronounced interfacial gaps. FLD showed the voids (white arrows) at the resin–dentin interface.

SEM observations of representative 24 h storage adhesive resin–dentin interfaces are shown in Figure 4. The 6m-stored interfaces could not be observed as all debonded when sliced. A well-defined hybrid layer between the dentin and resin composites and the resin tags inside dentin tubules could not be clearly observed in all specimens. Small gaps (black arrows/double-headed arrows) between resin composites and dentin could be detected in FIT, EXP, and FLD. Pronounced interfacial gaps (black arrows/double-headed arrows) between resin composites and dentin in VER and CON. FLD showed the voids (white arrows) at the resin–dentin interface.

### Water Sorption and Solubility

The WSP and WSL results were expressed as μg/mm^3^ and shown in Table 3. Concerning WSP, FLD (p = 0.000) and CON (p = 0.000) demonstrated the lowest values; and EXP, which presented the highest result, was significantly higher than VER (p = 0.005) but did not differ from FIT (p = 0.185). All five SACs showed higher WSP than the threshold (≤ 40 μg/mm^3^) specified in ISO 4049:2019. As for the WSL, VER exhibited the highest value (p = 0.000), which was higher than the WSL limit (≤ 7.5 μg/mm^3^) recommended by ISO 4049:2019. FLD exhibited the lowest WSL value (p = 0.000); and no statistically significant difference among FIT, EXP and CON was detected (p = 1.000).

**Table 3:** Mean and standard deviation (SD) of water sorption and solubility (μg/mm^3^) for each self-adhesive resin composite

Resin composite	Water sorption (SD)	Water solubility (SD)
FIT	65.79 (3.58)^a,b^	4.47 (0.90)^b^
EXP	69.44 (1.16)^a^	4.50 (0.92)^b^
FLD	40.03 (2.02)^c^	1.51 (0.47)^a^
VER	63.54 (1.06)^b^	8.03 (0.90)^c^
CON	44.77 (2.45)^d^	5.22 (0.27)^b^

Values followed by the same superscript letters in the same column indicate no statistically significant differences. The ISO 4049: 2019 standard was considered compliant when WSP ≤ 40 μg/mm^3^ and WSL ≤ 7.5 μg/mm^3^.

### Degree of Conversion

The degree of conversion of SACs was calculated according to the amplitude/intensity of the spectra peaks, expressed as %, and shown in Table 4. FIT and EXP showed significantly higher DC than FLD (p < 0.001), VER (p < 0.001), and CON (p < 0.001). VER showed significantly lowest DC (p < 0.001).

**Table 4:** Mean and standard deviation (SD) of the degree of conversion (%) according to the intensity/amplitude of the peaks for each resin composite

Resin composite	Intensity/Amplitude
FIT	93.86 (3.21) A
EXP	94.09 (0.94) A
FLD	79.83 (0.36) B
VER	71.10 (3.10) C
CON	84.08 (1.69) B

Different capital letters in the columns indicate statistically significant differences (p < 0.05).

## Discussion

The current study tested four commercial and one experimental SACs for their bonding properties to dentin, water sorption and solubility, and degree of conversion. After 6 m, only FIT and EXP had a significant decrease in bond strength. Therefore, the null hypothesis (i) that storage time would not affect the SACs’ bond strength to dentin was rejected. The bond strength to dentin of some SACs used in this study was previously examined using thermocycling with different regimens as an aging method and utilized distinct bond strength tests to enamel or dentin, such as tensile, shear, and microshear tests,^7,8,25,40^ with contradictory outcomes reported. From those studies, it is unclear if SACs’ adhesion to enamel and dentin is affected by any specific experimental condition and bond strength test. David et al. (2022) conducted a systematic review concluding that the bond strength of SACs is lower than that of conventional resin composites bonded to tooth substrates with an adhesive system.^11^ In addition, it has been reported that combining self-adhesive flowable composites with adhesive systems provides more effective bond strength.^10^

Only some studies have evaluated the differences between these SACs, without applying an adhesive system,^44^ and there is a lack of evidence exploring their direct interaction with tooth substrates. Thus, this study compared the microtensile bond strength of five different SACs strictly applied in dentin. The null hypothesis (ii) that different SACs would not affect bond strength to dentin was rejected as there was a statistically significant difference between the compared SACs. The bonding mechanism of SACs is through their self-etching or self-adhesive monomers (ie, 10-MDP and GPDM), which can chemically bond to hydroxyapatite but do not form a hybrid layer. The minimum bond strength of VER to dentin may be due to the unstable chemical bonding of GPDM to calcium in hydroxyapatite compared to other acidic functional monomers.^31,49^ Although 10-MDP-containing adhesives usually produce high bonding performance,^18^ CON did not present an expressive high bond strength in this study. It could be speculated that the poor bonding performance of CON might be due to the more complex composition of “all-in-one” materials, such as universal adhesives and SACs, than a two-step self-etch adhesive containing 10-MDP in its primer and bond resin.^49^ Also, VER and CON exhibited several pre-testing failures corroborated by previous studies.^35,36^ Although a “zero” value is attributed to pre-testing failures, these values represent very low bond strength, and do not mean non-existing bonding; the bond strength might be underestimated as it may require an amount of stress to produce the failure.^30^ In addition, the hybrid layer could not be observed at the SACs-dentin interface in the SEM images. Gaps between the SACs and dentin were commonly observed, depicting the limited ability of SACs to demineralize and penetrate the dentin matrix.^13^ The high percentage of pre-testing failure (Fig 2) and pronounced gaps (Fig 4) corroborate the low bond strength of VER and CON.

SACs’ WSP and WSL were tested for 7 days according to ISO 4049:2019 and there was a significant difference between materials (Table 3). The null hypothesis (iii) that water sorption and solubility would not be affected by different materials was rejected. All five SACs demonstrated WSP superior to ISO 4049:2019 standard, and only VER exhibited higher WSL than this standard. It has been reported that VER and FLD showed significantly higher long-term WSP than other resin composite types,^12,50^ corroborating our results regarding both SACs. The WSP of resin composites mainly depends on the polymer (monomer type, degree of conversion, and network characteristic),^20,50^ and the filler (morphology and dispersion in the polymer matrix).^20,50^ As the filler weight percentage increases, the polymer matrix contribution and water sorption decrease.^29^ The high WSP of the five SACs may be attributed to mobility demands of more diluent monomer and filler content.^50^ In addition, all the SACs contained the hydrophilic monomer HEMA. Although HEMA has been proven to improve the diffusivity of monomers into dentin substrate and achieve higher bond strength,^32^ it also leads to water sorption, promoting resin swelling, discoloration, and reduced mechanical strength over time.^46^

Moreover, the high WSP of SACs could also be the combined effect of the hydrophilic groups of the acidic functional monomer and polymeric monomers.^50^ Interestingly, FLD and CON exhibited lower WSP than the other SACs, which could be explained by the content of the functional monomer (4-MET and 10-MDP) and the polymeric monomer (TEGDMA). The aromatic group of 4-MET in FLD is hydrophobic and will adjust the acidity and hydrophilicity of the carboxyl group, which has poor solubility in water.^46^ Although the DC values were not the highest for FLD and CON, TEGDMA also presents a higher degree of conversion than UDMA and Bis-GMA.^23^ The long carbonyl chain of 10-MDP’s structure makes the monomer relatively hydrophobic,^46^ probably contributing to CON WSP similar to FLD. Leachable substances (monomer, filler, additive), and experimental conditions (immersion time, temperature, and solution) influence the WSL of resin composites.^2,3,50^ The leachable mass strongly depends on the degree of conversion of the polymerized monomers: the higher the degree of conversion, the lower the number of unreacted monomers, and the lower the solubility.^24,42^ Indeed, in this study, VER presented the highest WSL and the lowest DC. In addition, materials with high water sorption are not necessarily highly soluble and vice versa,^3^ which is confirmed by our results (Tables 3 and 4).

The null hypothesis (iv) that the degree of conversion would not be affected by different materials was rejected as there was a statistically significant difference between DC values (Table 4). The resin’s DC depends on the polymeric monomers’ chemical structure, the filler/resin ratio, and the polymerization conditions (sample thickness, temperature, and light-curing unit).^26,41^ This study determined the DC of SACs under the same polymerization conditions. Thus, the differences in the DC could be attributed to the SACs’ composition. Increasing the filler/resin ratio gradually reduced the degree of conversion,26 with the highest filler content VER among the five tested SACs exhibiting the lowest DC values (Tables 1 and 4). A high filler loading can inhibit free radical polymerization by electron transfer from the constituent oxides.^26^ Besides, VER is a Bis-GMA-based resin composite. Bis-GMA, present in VER and CON, is a highly viscous monomer (ie, relatively high molecular weight). It contains a rigid aromatic ring and strong hydrogen bonding in its molecule, resulting in a lower DC than UDMA and TEGDMA.^27,41,48^ The presence of UDMA in FIT, EXP, FLD, and CON confirms its high conversion rate. This monomer combines a relatively high weight, high concentration of double bonds, and low viscosity, achieving a higher DC than Bis-GMA. TEGDMA is a very low-viscosity monomer that can promote the movement of free radicals to form a flexible polymer network structure and enhance polymerization activity, thus increasing the DC,^27^ as shown by FLD and CON compared to VER. Overall, the combination and concentration of these monomers in the composition of each SAC, together with the filler presence and other components (eg, photoinitiators, stabilizers, and inhibitors), largely determine the degree of conversion to achieve the balance of properties and performance of the resin composites.

The commercially available material (FIT) and the experimental resin composite (EXP) from the same manufacturer were tested in this study. EXP presents a similar formulation of FIT, except for the filler. In the EXP, the S-PRG (present in FIT) was replaced by an equal volume of the silica to evaluate the differences caused by the filler. In this study, FIT and EXP obtained similar bond strength, water sorption and solubility, and degree of conversion. Therefore, based on our findings, substituting S-PRG filler for silica filler does not affect the properties tested. However, dental materials containing S-PRG fillers exhibited buffering capacity, inhibited demineralization, and promoted remineralization, and can be recommended for clinical applications.^33^

Thus, based on the outcomes of our research, the SACs still present limitations in the assessed physical-mechanical properties, which still require improvement. Additional laboratory studies and well-designed randomized clinical trials are needed before recommending a broad clinical use of the SACs.

## Conclusions

Within the limitations of the present study, the following conclusions can be drawn:

1. The bond strength to dentin (at both 24 h and 6 m) varied among the SACs. Vertise Flow and Constic exhibited the least satisfactory bonding performance after both storage periods.
2. Long-term water storage (6 m) weakened the bond strength to dentin of FIT SA F03 and the experimental resin composite.
3. All five SACs exhibited higher water sorption than those values recommended by the ISO 4049:2019 standard.
4. Except for Vertise Flow, the other SACs presented solubility values within the standards recommended by ISO 4049: 2019 standard.
5. Among the investigated SACs, FIT SA F03 and the experimental resin showed the highest degree of conversion.

### Clinical Relevance

SACs are highly attractive since they claim to eliminate the need for the bonding procedure. Nevertheless, these materials still possess constraints related to their physical and mechanical properties, especially their long-term bond strength to dentin. Therefore, their application should be considered cautiously, and further clinical trials are necessary to evaluate SACs’ long-term performance concerning additional clinically relevant properties such as marginal adaptation, marginal staining, fracture and retention, postoperative sensitivity, and recurrence of caries for each use recommended by the manufacturers.
